# Photostability studies on (±)-tramadol in a liquid formulation

**DOI:** 10.1186/s40780-014-0003-2

**Published:** 2015-02-05

**Authors:** Manabu Suno, Hidenori Ichihara, Takahiro Ishino, Kento Yamamoto, Yuta Yoshizaki

**Affiliations:** Department of Oncology Pharmaceutical Care & Sciences, Graduate School of Medicine, Dentistry and Pharmaceutical Sciences, Okayama University, 2-5-1, Shikata-cho, Kita-ku, Okayama, 700-8558 Japan

**Keywords:** Tramadol, Photodegradation, Liquid formulation, Photostability

## Abstract

**Background:**

Tramadol ((±)-TRA) is recommended for the treatment of mild to moderate cancer pain by the World Health Organization. An oral liquid formulation of (±)-TRA is preferable for patients with a compromised swallowing function. However, the stability of (±)-TRA in aqueous solution has yet to be determined in a clinical setting. The aim of this study was to evaluate the photostability of (±)-TRA in aqueous solution in a clinical setting.

**Methods:**

We improved high performance liquid chromatography (HPLC) method for the enantiomeric separation of (±)-TRA, and then the (±)-TRA concentration was determined using HPLC method. We investigated the photodegradation of (±)-TRA in an aqueous solution irradiated with ultraviolet (UV) light: UV-A, UV-B, and UV-C. We also evaluated the stability of liquid formulations of (±)-TRA in a clinical setting by keeping (±)-TRA aqueous solution in normal dispensing bottles and in light-shading dispensing bottles under conditions of both sunlight and diffused light in a room. Samples were collected sequentially over time.

**Results:**

(±)-TRA in aqueous solution was degraded the most rapidly when irradiated with UV-C, but was not affected by irradiation with UV-A. No significant difference was observed in the photodegradation behavior of (+)-TRA and (−)-TRA with UV-A, UV-B, and UV-C irradiation. The residual percentages of (±)-TRA were 94.6-104.3% after 14 days in the presence of either sunlight or diffused light in a room, with or without protection from light.

**Conclusions:**

These results demonstrated the stability of (±)**-**TRA in aqueous solution to both sunlight and diffused light in a room. Therefore, liquid formulations of TRA are preserved at room temperature for up to 2 weeks, with or without protection from light. Our results provide additional treatment options with tramadol for pain control.

## Background

Tramadol hydrochloride (TRA), (1*RS*, 2*RS*)-2-[(dimethylamino) methyl]-1-(3-methoxyphenyl) cyclohexanol hydrochloride, is categorized as a weak opioid on the second step of the three-step analgesic ladder for cancer pain relief by the World Health Organization (WHO). It has been recommended for the treatment of mild to moderate cancer pain [1]. (±)-TRA achieves its analgesic effects by increasing both serotonin and noradrenaline concentrations in the synaptic cleft of neurons in the central nervous system [2-4]. (+)-TRA mainly inhibits serotonin reuptake, while (−)-TRA chiefly blocks noradrenaline reuptake. TRA is available as capsules, injections, and acetaminophen-containing agents, and is generally used to relieve pain associated with cancer and post-operative pain [5]. According to the WHO, analgesics for cancer should be administered orally [1]. Oral liquid formulations are preferable for patients with a compromised swallowing function, such as the elderly or cancer patients, including those with laryngeal and oropharyngeal cancers [6-11].

The photodegradation of (±)**-**TRA by ultraviolet (UV) irradiation is known to follow a first-order reaction [12-14]. However, the stability of (±)-TRA in aqueous solution in the presence of either sunlight or diffused light has not yet been examined in detail. The storage environment has the potential to have marked effects on the quality of TRA liquid formulations.

The aim of this study was to evaluate the photostability of (±)-TRA in aqueous solution in a clinical setting. We investigated the photodegradation behavior of (±)-TRA in aqueous solution irradiated with UV-A, UV-B, and UV-C. We also evaluated the stability of (±)**-**TRA in aqueous solution to light when stored in two conditions, involving sunlight and diffused light in a room for 2 weeks.

## Methods

### Chemicals and materials

Tramadol (TRA) capsules (tramal® capsules 25 mg; Nihon Shinyaku, Japan) were obtained for the preparation of (±)-TRA in aqueous solution. TRA hydrochloride (LKT Laboratories, MN) was used as the reference standard. Propranolol (Tokyo Kasei, Japan) was used as the internal standard. Acetonitrile and methanol (Merck, Germany) were products graded for high performance liquid chromatography (HPLC). Ammonium formate and diethylamine (Sigma-Aldrich, MO) were obtained for HPLC. Ultrapure water (Wako, Japan) was used to dissolve ammonium formate. Ultraviolet (UV) irradiation was carried out with a UV-A lamp (FL15BL, 15 W, dominant wavelength 352 nm,wavelength range 300–400 nm; Toshiba, Japan), a UV-B lamp (GL15E, 15 W, dominant wavelength 306 nm,wavelength range 280–320 nm; Sankyo, Japan), and a UV-C lamp (GL-15, 15 W, dominant wavelength 253.7 nm; Toshiba, Japan). A fluorescent lamp (FHF32EX-N-H, 32 W, wavelength range 400–700 nm; Panasonic, Japan) was used for diffuse light.

### Preparation of (±)-TRA in aqueous solution

(±)-TRA in aqueous solution was prepared by emptying the contents of four TRA capsules into 40 mL of Milli-Q and filtering the solution with filter paper (Advantec, Japan). Normal dispensing bottles (NDBs) (M.I-chemical, Japan) were used for (±)-TRA in aqueous solution under the conditions of UV irradiation, and light-shading dispensing bottles (LSDBs) (M.I-chemical, Japan) as well as NDBs were used for (±)-TRA in aqueous solution under the conditions of both sunlight and diffused light in a room.

### Assay for (±)-TRA

(±)-TRA was resolved optically. A peak height ratio analysis was employed to calculate the concentration of (+)-TRA and (−)-TRA using (+)-propranolol and (−)-propranolol as an internal standard, respectively. HPLC was performed with a UV detector (UV-2075; Jasco, Japan), HPLC pump (PU-2080; Jasco, Japan), and an analytical column (CHIRALCEL OD-RH, 5 μm, 4.6 mm × 150 mm i.d.; Daicel, Japan) in combination with a guard column (CHIRALCEL OD-RH, 5 μm, 4.6 mm × 10 mm i.d.; Daicel, Japan), and with a mobile phase of 5 mM ammonium formate with 0.1% diethylamine in water/acetonitrile (57:43, v/v), at 0.8 mL/min, 265 nm, and room temperature. The run time was 30 min.

Ten μL of the collected sample was pipetted into a 1.5 mL polypropylene tube, and 480 μL of the mobile phase was added and vortexed for 10 sec. Ten μL of (±)-propranolol as an internal standard in methanol (1 μg/μL) was added to the mixture and vortexed for 10 sec, and then a 10 μL aliquot was injected onto the HPLC system.

### Degradation of (±)-TRA in aqueous solution by UV-A, UV-B, and UV-C irradiation

(±)-TRA in aqueous solution was irradiated with UV-A, UV-B, and UV-C to analyze the photodegradation of (±)-TRA in aqueous solution. The samples (each 300 μL) were collected before and at 10, 20, and 30 min, 1, 2, 4, 6, 8, 24, and 48 h after UV-A, UV-B, and UV-C irradiation. The samples were removed from the light and stored at 5°C until analysis. The first-order reaction rate constant (*k*) of (±)-TRA degradation in aqueous solution by UV-A, UV-B, and UV-C irradiation was calculated.

### Photostability of (±)-TRA in aqueous solution in a clinical setting

NDBs and LSDBs were placed at a distance of 15 cm from a window and maintained at room temperature (20-25°C). The samples were irradiated by sunlight that passed through the window and used to evaluate the photostability of (±)-TRA in aqueous solution under the condition of sunlight in a room. The measurement samples (each 300 μL) were collected at 0, 1, 2, 3, 5, 7, and 14 days after exposure to these conditions. The samples were removed from the light and stored at 5°C until analysis.

NDBs and LSDBs were placed at room temperature (20-25°C), and darkness and irradiation with a fluorescent lamp light were alternated every 12 h to evaluate the photostability of (±)-TRA in aqueous solution under the condition of diffused light in a room. The samples (each 300 μL) were collected at 0, 2, 4, 7, and 14 days after exposure to these conditions. The samples were removed from the light and stored at 5°C until analysis.

### Statistical analysis

Statistical analyses were performed using SPSS (SPSS® Statistics 22.0; IBM, Japan). The residual percentage of (±)-TRA in aqueous solution was expressed as the mean ± standard deviation (SD). In the experiment to examine the photostability of (±)-TRA in aqueous solution in a clinical setting, repeated measures ANOVA was used to compare the residual percentages of (±)-TRA in aqueous solution at day 7 and 14 with that at day 0. The unpaired *t*-test was performed to show the *P*-value between dispensing bottles, light conditions, and isomers on day 14 in the comparison of residual percentages of (±)-TRA in aqueous solution. In all analyses, *P* <0.05 was considered significant.

## Results

### Chromatographic separation

**Figure 1:**
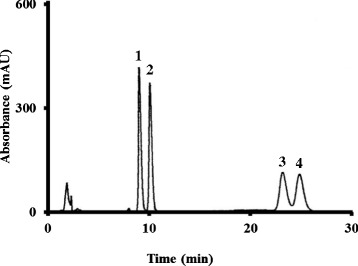
**HPLC chromatogram of (±)-tramadol in aqueous solution.** Peak 1; (+)-tramadol, 2; (−)-tramadol, 3; (+)-propranolol, and 4; (−)-propranolol.

(±)-TRA and (±)-propranolol separated well using the HPLC conditions described above (Figure 1). The retention times of (+)-TRA, (−)-TRA, (+)-propranolol, and (−)-propranolol in aqueous solution were 8.5, 9.5, 21.3, and 24.0 min, respectively.

### Calibration curve

The standard curves were expressed as the regression equation of a straight-line, y = ax + b, where y is the concentration, a is the slope, x is the peak height ratio against (±)-propranolol, and b is the y-axis intercept, by the weighted liner regression analysis. The calibration curve, which covered a concentration range of 5.0-100.0 ng/μL of (±)-TRA exhibited linearity and had a coefficient of correlation ((+)-TRA: y =0.0822x +0.0896, *r* =0.999, *P* <0.01, and (−)-TRA: y =0.0748x +0.0697, *r* =0.999, *P* <0.01). The lower quantification limit of (±)-TRA was 5 ng/μL (signal to noise; S/N >3).

### Accuracy and precision

The accuracy and precision of the methods used was confirmed at 5 different concentrations of (±)-TRA. The method was reproducible with coefficients of validation (CV) less than 5% for both intra- and inter-day variability (Table 1). These values are in the acceptable range of the US Food and Drug Administration guidance [15].

**Table 1:** **Accuracy and precision study on the (±)-tramadol assay**

**Nominal**		**Run-to-run assay**			**Day-to-day assay**		
**concentration (ng/μL)**		**Detected (ng/μL)**	**Accuracy (%)**	**CV (%)**	**Detected (ng/μL)**	**Accuracy (%)**	**CV (%)**
(+)-tramadol	5.0	5.2 ± 0.1	103.4	1.91	5.2 ± 0.1	104.3	1.90
	10.0	9.5 ± 0.3	96.0	3.29	9.6 ± 0.3	94.9	3.16
	25.0	26.7 ± 0.4	103.9	1.56	26.0 ± 0.6	104.0	2.89
	50.0	50.4 ± 0.9	100.0	1.70	50.0 ± 0.8	100.8	1.41
	100.0	99.5 ± 2.2	100.3	2.20	100.3 ± 2.8	99.5	2.80
(-)-tramadol	5.0	4.9 ± 0.2	94.8	4.10	4.7 ± 0.2	98.0	4.30
	10.0	9.5 ± 0.3	96.5	2.79	9.4 ± 0.2	94.5	2.25
	25.0	26.1 ± 0.9	104.5	3.44	26.1 ± 0.3	104.4	1.18
	50.0	50.7 ± 0.9	99.1	1.82	49.6 ± 0.4	101.4	0.88
	100.0	100.6 ± 2.1	101.2	2.10	101.2 ± 2.8	100.6	2.44

Data are expressed as the mean ± SD of 6 analyses.

CV; coefficient of variation.

### Photodegradation of (±)-TRA in aqueous solution by UV irradiation

**Figure 2:**
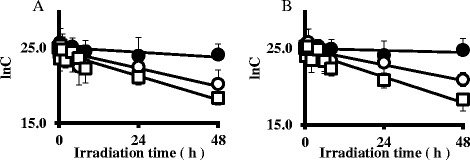
**Plot of ln C vs time for the detection of (±)-tramadol in aqueous solution irradiated by UV-A, UV-B, and UV-C. A**: (+)-tramadol in aqueous solution irradiated by UV-A (●), UV-B (○), and UV-C (□). **B**: (−)-tramadol in aqueous solution irradiated by UV-A (●), UV-B (○), and UV-C (□).

(±)-TRA in aqueous solution was degraded when irradiated with UV-B and UV-C, but was not affected by irradiation with UV-A. No significant difference was observed in the photodegradation behavior of (+)-TRA or (−)-TRA exposed to UV-A, UV-B, and UV-C irradiation (Figure 2).

The *k* values of (+)-TRA irradiated with UV-A, UV-B, and UV-C were 1.00 × 10^−3^, 4.50 × 10^−3^, and 6.20 × 10^−3^ (h^−1^), respectively. The *k* values of (−)-TRA irradiated with UV-A, UV-B, and UV-C were 0.40 × 10^−3^, 3.70 × 10^−3^, and 7.10 × 10^−3^ (h^−1^), respectively (Table 2). These results indicated that (±)-TRA in aqueous solution was degraded the most rapidly by UV-C irradiation.

**Table 2:** **First-order degradation rate constants for (±)-tramadol in aqueous solution exposed to UV irradiation**

		**UV-A lamp**		**UV-B lamp**		**UV-C lamp**	
	**(** ***n*** **)**	***k*** **(h** ^**−1**^ **)**	***r***	***k*** **(h** ^**−1**^ **)**	***r***	***k*** **(h** ^**−1**^ **)**	***r***
(+)-tramadol	(5)	1.00 × 10^−3^	−0.237	4.50 × 10^−3^	−0.881	6.20 × 10^−3^	−0.953
(−)-tramadol	(5)	0.40 × 10^−3^	−0.127	3.70 × 10^−3^	−0.921	7.10 × 10^−3^	−0.970

*k*; first-order reaction rate constant, *r*; correlation coefficient.

### Photostability of (±)-TRA in aqueous solution in a clinical setting

**Figure 3:**
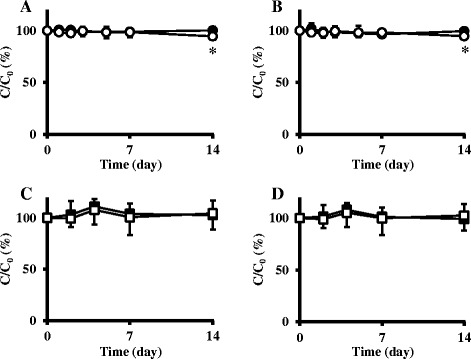
**Time courses of the residual percentages of (±)-tramadol in aqueous solution contained in normal dispensing bottles (NDBs) and in light-shading dispensing bottles (LSDBs) under the conditions of both sunlight and diffused light in a room. A**: (+)-tramadol in aqueous solution contained in NDBs (○) and in LSDBs (●) under the conditions of sunlight in a room. **B**: (−)-tramadol in aqueous solution contained in NDBs (○) and in LSDBs (●) under the conditions of sunlight in a room. **C**: (+)-tramadol in aqueous solution contained in NDBs (□) and in LSDBs (■) under the conditions of diffused light in a room. **D**: (−)-tramadol in aqueous solution contained in NDBs (□) and in LSDBs (■) under the conditions of diffused light in a room. The plots represent the mean ± SD. The residual percentages of (±)-tramadol in aqueous solution at day 7 and 14 were compared with day 0 by the repeated measures ANOVA. A significant reduction in the residual percentages of (±)-TRA was observed between day 0 and 14 in NDBs under the condition of sunlight in a room (* *P* <0.05).

(±)-TRA in NDBs was slightly degraded under the condition of sunlight in a room (Figure 3). No significant difference was observed in the residual percentages of (±)-TRA between day 0 and 7 in NDBs under these conditions. On the other hand, a significant reduction in the residual percentages of (±)-TRA was observed between day 0 and 14 in NDBs under these conditions ((+)-TRA; *P* <0.05, and (−)-TRA; *P* <0.05). (±)-TRA in NDBs was not degraded under the condition of diffused light in a room (Figure 3). No significant difference was observed in the concentration of (±)-TRA in NDBs between day 0 and 7 or between day 0 and 14 under these conditions, respectively. The residual percentages of (±)-TRA in both NDBs and LSDBs were 94.6-104.3% after 14 days under both conditions of sunlight and diffused light in a room. No significant difference was observed in the residual percentages of (±)-TRA between either the dispensing bottles or light conditions (Table 3-A). No significant difference was also found between the residual percentages of (+)-tramadol and (−)-tramadol (Table 3-B).

**Table 3:** **Residual percentages of (±)-tramadol in aqueous solution in a clinical setting at day 14**

**(A) Comparison of dispensing bottles and light conditions**									
				**Residual percentages (C/C**_**0,**_ **%)**					
		**(** ***n*** **)**		**NDBs**		**LSDBs**		***P*** **-value**	
(±)-tramadol (Sunlight)		(5)		94.6 ± 3.1	*P* =0.29	99.9 ± 2.8	*P* =0.90	0.20	
(±)-tramadol (Diffused light)		(5)		103.4 ± 15.9		100.9 ± 15.3		0.82	
**B) Comparison of (+)-tramadol and (−)-tramadol**									
		**Residual percentages (C/C**_**0,**_ **%)**							
		**Sunlight**				**Diffused light**			
	**(** ***n*** **)**	**NDBs**		**LSDBs**		**NDBs**		**LSDBs**	
(+)-tramadol	(5)	94.6 ± 3.4	*P* =0.97	100.2 ± 3.4	*P* =0.75	104.3 ± 15.6	*P* =0.86	102.6 ± 14.4	*P* =0.75
(−)-tramadol	(5)	94.7 ± 2.4		99.6 ± 1.7		102.4 ± 14.5		99.2 ± 14.4	

NDBs: normal dispensing bottles.

LSDBs: light-shading dispensing bottles.

The unpaired *t*-test was performed to calculate the *P*-value between the dispensing bottles, light conditions, and isomers.

## Discussion

The photodegradation of (±)-TRA in aqueous solution proceeded the most rapidly when exposed to UV-C irradiation whereas UV-A irradiation had no effect. No significant difference was observed in the photodegradation behavior of (+)-TRA and (−)-TRA with each type of UV irradiation.

Many pharmaceutical compounds in solution requiring protection from light are degraded not only by UV-B and UV-C irradiation, but also by UV-A irradiation [16-20]. Such UV-sensitive pharmaceutical compounds include amiodarone, dapsone, dexametazone, lacidipine, lercanidipine, naproxen, nifedipine, and pitavastatin [16-20]. Pitavastatin in solution is very sensitive to UV-A irradiation, and the *k* of pitavastatin in solution when irradiated with UV-A is approximately 1.3 h^−1^, which is considerably larger than the *k* of (+)-TRA and (−)-TRA in our study [20]. We found that (±)-TRA in aqueous solution was not degraded by irradiation with UV-A, confirming the stability of (±)-TRA in aqueous solution to UV-A.

The mean residual percentages of (±)-TRA in aqueous solution after 2 weeks in a clinical setting ranged from 94.6-104.3%, although a significant reduction was observed between day 0 and 14 under the condition of sunlight in a room (Figure 3-A and -B). The range of the mean residual percentages satisfied the United States Pharmacopeia uniformity of content for liquid orals that range from 85-115% [21]. These results demonstrate the stability of liquid formulations of TRA for 2 weeks in a clinical setting. Our results indicate that the mean residual percentages of (±)-TRA in aqueous solution under the condition of sunlight in a room ranged from 94.6-100.2%. UV contained within sunlight has been categorized into 3 main types of UV-A, UV-B, and UV-C [22]. The percentages of UV-A and UV-B contained within sunlight reaching the ground are approximately 5.3% and 0.14%, respectively, while UV-C does not reach the ground due to its complete absorption in the atmosphere [23,24]. Therefore, the photodegradation of pharmaceutical compounds under the condition of sunlight in a room was thought to be attributed to mainly UV-A. Our results indicated that the photodegradation of (±)**-**TRA in aqueous solution was not caused by UV-A irradiation, which explains the stability of (±)-TRA in aqueous solution under the condition of sunlight in a room. The mean residual percentages of (±)-TRA in aqueous solution under the condition of diffused light in a room ranged from 99.2-104.3%. The wavelength region of the fluorescent lamp used under the condition of diffused light was longer than UV-A, and the photodegradation of (±)-TRA in aqueous solution would thus not be caused by the fluorescent lamp irradiation. These results suggest that liquid formulations of TRA are stable at room temperature for 2 weeks, with or without protection from light.

This study evaluated the photostability of (±)-TRA in aqueous solution in a clinical setting. We showed stability of the liquid formulations of TRA for 2 weeks. It should be noted that this study has the limitation that the microbiological safety was not assessed. Therefore, further evaluation of the microbiological safety of (±)-TRA in aqueous solution will be necessary.

## Conclusions

Our results indicated that the photodegradation of (±)-TRA in aqueous solution proceeded the most rapidly by UV-C irradiation whereas UV-A irradiation had no effect on its stability. The photodegradation of (±)-TRA in aqueous solution did not proceed in a clinical setting, which confirmed the stability of the TRA liquid formulations. Our results demonstrate the stability of liquid formulations of TRA at room temperature for 2 weeks, and provide additional treatment options to relieve pain in patients with a compromised swallowing function.

## Abbreviations

HPLC: High performance liquid chromatography
*k*: First-order reaction rate constant
LSDBs: Light-shading dispensing bottles
NDBs: Normal dispensing bottles
TRA: Tramadol
UV: Ultraviolet
WHO: World Health Organization
